# Association of obstructive sleep apnea with non-alcoholic fatty liver disease in patients with obesity: an observational study

**DOI:** 10.1007/s40519-021-01182-9

**Published:** 2021-04-03

**Authors:** Silvia Bettini, Roberto Serra, Roberto Fabris, Chiara Dal Prà, Francesca Favaretto, Francesca Dassie, Claudio Duso, Roberto Vettor, Luca Busetto

**Affiliations:** 1 Department of Medicine, Internal Medicine 3, University of Padova, Via Giustiniani 2, 35128 Padova, Italy; 2 Center for the Study and the Integrated Management of Obesity, Padova University Hospital, Padova, Italy

**Keywords:** Obstructive Sleep Apnea Syndrome, Non-Alcoholic Fatty Liver Disease, FIB-4, Obesity, Fibrosis

## Abstract

**Purpose:**

Obstructive Sleep Apnea (OSA) is associated with the presence and severity of Non-Alcoholic Fatty Liver Disease (NAFLD). We aimed to investigate the relationship between the severity of OSA and NAFLD and to recognize a polysomnographic parameter correlated with progression of fibrosis, determined by a non-invasive score of liver fibrosis, FIBrosis-4 index (FIB-4), in patients affected by severe obesity and OSA.

**Methods:**

We enrolled 334 patients (Body Mass Index, BMI 44.78 ± 8.99 kg/m^2^), divided into classes according to severity of OSA evaluated with Apnea Hypopnea Index (AHI): OSAS 0 or absent (17%), mild OSA (26%), moderate OSA (20%), severe OSAS (37%). We studied anthropometric, polysomnographic, biochemical data and FIB-4. A multiple regression model was computed to identify a polysomnographic independent predictor of FIB-4 among those parameters previously simple correlated with FIB-4.

**Results:**

The severity of OSA was associated with a decrease in High-Density Lipoprotein–cholesterol (HDL) and an increase in BMI, triglycerides, Homeostasis model assessment insulin-resistance index (HOMA), transaminases and FIB-4. FIB-4 correlated with sex, age, BMI, AHI, mean percentage oxyhaemoglobin (meanSaO2%), number of desaturations, platelets, transaminases, HDL, triglycerides and HOMA. The only variables independently related to FIB-4 were sex, BMI, triglycerides and meanSpO2 (*r* = 0.47, AdjRsqr = 0.197).

**Conclusion:**

MeanSpO2% represented an independent determinant for the worsening of FIB-4 in patients with severe obesity and OSA. Hence, it could hypothesize a clinical role of meanSaO2% in recognizing patients with obesity and OSA and higher risk of developing advanced fibrosis and, thus, to undergo further investigation.

**Level III:**

Evidence obtained from well-designed cohort analytic studies.

## Introduction

In the last decades we observed a pandemic increase in the prevalence of metabolic diseases such as obesity, Type 2 Diabetes Mellitus (T2DM) and non-alcoholic fatty liver disease (NAFLD) [1–3] and, on the other hand, of Obstructive Sleep Apnea (OSA) [4–6]. NAFLD is an emerging disease in western countries, representing about 75% of all chronic liver diseases and one of the most worrying complications of obesity. NAFLD is associated with progressive insulin-resistance [1–3] and, on the other hand, insulin-resistance predicts NAFLD improvement in patients undergoing a dietary intervention for weight loss [7]. NAFLD includes different clinical conditions: Non-Alcoholic Fatty Liver (NAFL), histologically characterized by simple liver steatosis, and Non-Alcoholic Steato-Hepatitis (NASH) that covers a broad spectrum of complications, from fibrosis to cirrhosis to hepatocellular carcinoma [2, 3]. It was estimated that the global prevalence of NAFLD is around 25% [1] and that it grows consensually with related metabolic diseases, in particular obesity [2, 3]. Indeed, several genetic modifiers of NAFLD have been identified and the best-characterized genetic association is with PNPLA3, found also in patients without insulin-resistance [2].

OSA is characterized by repetitive episodes of partial or total loss of respiratory airflow during sleep with the collapse of the upper airway during inspiration and is accompanied by strenuous breathing [4]. In general population, OSA prevalence is approximately 10–17% in men and 3–9% in women [6], whereas the prevalence of OSA in people with obesity is about 48% in men and 38% in women [8]. Visceral obesity is related to OSA presence and severity [9, 10] and OSA is associated with an increased cardiovascular risk, probably driven by changes in glucose and lipid metabolism [11]. Pathophysiological mechanisms by which obesity promotes OSA are not only a simplistic mechanical impact that the expansion of adipose tissue has on the airways. Thus, it has been suggested that the expansion of the visceral adipose tissue, the alteration of the adipocytokines, the increase in circulatory levels of inflammatory mediators such as Tumor Necrosis Factor (TNF)α, interleukin (IL)-6, IL-1, IL-1β and nuclear factor kB (NF-kB) and the recruitment of immune cells determine a chronic low-grade inflammation which is implicated in the appearance and aggravation of OSA [9, 11–17]. The association between obesity and lung disease is not limited to OSA. Covid-19 respiratory syndrome is a current example of how adipose tissue disfunction can lead to lung injury [18].

In the same way, NAFLD positively correlates with visceral fat depot and obesity comorbidities (such as T2DM and OSA) worsen the histopathological features of NAFLD [2, 3, 19]. As for OSA, adipocytokines play an important role in the pathogenesis and progression of NAFLD, together with a direct effect of insulin resistance as well as oxidative stress [20–24].

Identifying patients with the greatest risk of evolution of NAFLD to NASH represents a challenge for clinicians. It is worth to note that fibrosis is the predictive criterion for a poor prognosis [2, 3, 25, 26] and, although liver biopsy is considered the diagnostic gold standard to detect fibrosis, it is expensive, invasive and therefore not suitable for general population studies [27]. Indeed, liver biopsy is also affected by sampling bias, providing only a very small sample with the risk of not to be representative for the amount of hepatic fibrosis in the whole liver [28]. Thus, it is recommended to calculate non-invasive fibrosis biomarkers and scores in patients with NAFLD, to identify patients at higher risk to undergo to liver biopsy [2, 3]. Among these, FIBrosis-4 index (FIB-4), NAFLD fibrosis scores, alanine aminotransferase (ALT)/aspartate aminotransferase (AST) ratio, Aspartate aminotransferases-to-Platelet Ratio Index (APRI) have been validated under several conditions of chronic liver disease and in the NAFLD, and FIB-4 especially, demonstrating sufficient sensitivity and specificity to be used in clinical practice [26, 29–31].

A growing amount of evidence has correlated OSA with the presence of NAFLD and it has been demonstrated a direct association between the severity of the former and the progression of the latter [32]. Thus, OSA patients should be screened for the presence and severity of NAFLD [32]. Non-invasive biomarkers and scores could become a screening tool to identify patients to be referred to liver biopsy and therefore to prevent NAFLD progression in patients living with obesity and OSA. It was suggested that the chronic intermittent hypoxia present in OSA triggers and increases the accumulation of triglycerides (TG), low-grade inflammation, tissue necrosis and liver fibrosis [24, 33]. However, pathophysiological mechanisms by which intermittent hypoxia lead to simple liver steatosis to the development of fibrosis and cirrhosis are still unclear. Therefore, the aim of this study was firstly, to analyze the association between OSA and NAFLD in patients with severe obesity and to investigate a possible direct correlation between polysomnographic parameters and a non-invasive score of liver fibrosis, and secondly to recognize an independent determinant of OSA to predict NAFLD progression.

## Material and methods

### Patients

Three hundred and thirty-four Caucasian patients with obesity (Boby Mass Index, BMI ≥ 30 kg/m^2^) were enrolled at the Centre for the Study and Integrated Treatment of Obesity, Padua University Hospital, in the period 2013–2019. Patients underwent a multi-disciplinary evaluation according to a standard clinical protocol and a complete medical history was taken regarding eating, physical activity, smoking and drinking habits, drugs, past and current medical conditions. Indeed, these patients underwent to polysomnography according to the suspect of OSA on the basis of clinical evaluation and a validated questionnaire (the Epworth Sleepiness Scale, ESS). These subjects were also eligible for bariatric surgery and, thus, provided the result of a simple abdominal ultrasound scan (US), as pre-surgery exam according to a standard clinical protocol. All US confirmed the presence of fatty liver. Specific exclusion criteria for this study were previous bariatric surgery, continuous positive airway pressure therapy (CPAP), unavailability of all clinical anamnestic, anthropometric, biochemical and polysomnographic parameters, excessive alcohol consumption (daily alcohol consumption ≥ 30 g for men and ≥ 20 g for women [2]), non-metabolic liver disease (i.e. viral or autoimmune hepatitis, genetic hemochromatosis, Wilson disease), a current infection and use of hepatotoxic drugs. Laboratory exams and polysomnography were scheduled before bariatric surgery closely in time not greater than two months, and with not significant difference in weight.

All anthropometric measurements were taken with subjects wearing only light clothes without shoes. Height was measured to the nearest 0.01 m. Body weight was determined to the nearest 0.1 kg using a calibrated balance beam scale.

All subjects gave written informed consent in accordance with the Declaration of Helsinki. The protocol was approved by the ‘Padua Ethical Committee for Clinical Research’ (2892P, 10/06/2013).

### Biochemical assessment

For each patient, all blood tests were performed in the morning, after 8 h of fasting. All biochemical blood analyses have been performed with standard diagnostic kit according to WHO First International Reference Standard: fasting plasma glucose (FPG) (Glucose HK Gen.3, Roche Diagnostic, Indianapolis, IN, USA), basal insulin, IL-6, TNFα (IMMULITE 2000 Immunoassay, Siemens Healthcare GmbH, Erlangen, Germany), high-sensitivity C-Reactive Protein (hs-CRP) (CardioPhase High Sensitivity C-Reactive Protein, Siemens Healthcare, Erlangen, Germany), Leptin (Leptin-RIA-CT, Mediagnost, Reutlingen, Germany), vitamin D3 (Diasorin Liaison XL LAS, Saluggia, Italy). Platelets were measured by flow cytometry (Sysmex Europe GmbH, Norderstedt, Germany), lipid profile [(total cholesterol (TC), High-Density Lipoprotein–cholesterol (HDL–cholesterol) and TG] by spectrophotometer (Cobas 8000, Roche Diagnostic, Indianapolis, IN, USA). Low Density Lipoprotein–cholesterol (LDL–cholesterol) was calculated according to Friedewald [34]. ALT, AST, gamma glutamyltransferase (GGT) levels were assayed by enzymatic method with the addition of pyridoxal-5-phosphate in compliance with IFCC reference methods [35]. Leptin, IL-6 and TNF-α were not available for 63 patients and hs-CRP for 124 patients. Missing data were homogeneously distributed into OSA categories.

The insulin-resistance index was indirectly estimated using the homeostasis model assessment (HOMA) as follow: [fasting serum insulin (μU/ml) x fasting plasma glucose (mmol/l)]/22.5 [36].

### Non-invasive fibrosis score

FIB-4 was calculated using the following formula: [age (years) x AST]/[platelet counts (× 10^9^/l) x ALT^1/2^] [26, 30]. A low cutoff point (< 1.30) and a high cutoff point (> 2.67) best discriminate between the absence and presence of advanced fibrosis, respectively [30].

### Polysomnographic evaluation

The polysomnographic analysis was performed using the SOMNOtouchTM® NIBP device (SOMNOmedics Italia, Bellusco, Italy) and a complete cardiorespiratory night monitoring was carried out for at least 8 h. During polysomnography peripheral, blood oxygen saturation, respiratory flow in the upper airway, respiratory movements of the chest and abdomen, snoring phases, the position of the patient, blood pressure and electrocardiogram with acquisition of peripheral signals were recorded. The recorded layout and thus the criterion for assigning hypopneas scoring was automatically analyzed by the DOMINO software®, validated by SOMNOmedics, and subsequently it was manually validated by an expert operator according to the most recent American Academy of Sleep Medicine (AASM) criteria [37]. The anthropometric data included age, height and weight. The polysomnographic data encompassed Apnea Hypopnea Index (AHI), minimum percentage oxyhaemoglobin saturation (mSaO2%), mean percentage oxyhaemoglobin saturation (meanSaO2%), percentage of time with oxyhaemoglobin saturation percentage less than 90 (TS < 90%) and number of desaturations with oxyhaemoglobin saturation < 90% (nSaO2 < 90%).

### Statistical analysis

Statistical analyses were performed using the SigmaPlot v.14 (Systat Software, Adalta, Arezzo, Italy). All variables were tested by normal Test (Shapiro–Wilk test) and Equal Variance Test (Brown-Forsythe). One Way Analysis of Variance was used when Normality Test and Equal Variance Test have been passed (data are presented as mean values ± standard deviations) or, if not, with the Kruskal–Wallis One Way Analysis of Variance on Ranks (data are presented as median value (25th–75th percentile)). Chi-square test was carried out for categorical variables. Pearson’s correlation coefficient (*r*) and the relative *p* values were calculated to analyze simple linear correlations between two variables. A multiple regression model with adjustment for presence of diabetes, hypertension and dyslipidemia was computed with FIB-4 as the dependent variable and the variables found to be simple correlated with FIB-4 as independent variables. Variant Inflation Factor (VIF) was considered as measure of multicollinearity and a value greater than 4 was considered as cut off for excluding variables from the analysis. In all analyses, the *p* values were two-sided and a *p* value lower than 0.05 was considered statistically significant.

## Results

Three hundred and thirty-four patients with severe obesity had a mean age of 48 ± 12 years (range 18–79), 158 were male (47%) and the mean BMI was 44.78 ± 8.99 kg/m^2^ (IQR 38.5–49.9). Patients with hypertension were 204 (61.1%); dyslipidemia, defined according to recent guidelines [38], was present in 197 patients (59%). According to the American Diabetes Association [39], 101 patients were affected by T2DM (30.2%), 54 (16.2%) had prediabetes (impaired fasting glycemia or/and impaired glucose tolerance at the oral glucose tolerance test), 179 (53.6%) had a normal glycemia. Clinical, laboratory and polysomnographic evaluations of the 334 patients, divided in relation to OSA severity (AHI), are reported in Table 1. Fifty seven (17%) of the patients were classified as no OSA (AHI < 5), 88 (26%) as mild OSA (AHI 5–14), 65 (20%) as moderate OSA (AHI 15–29) and 124 (37%) as severe OSA (AHI ≥ 30). As expected, all polysomnographic parameters (mSaO2%, meanSaO2%, nSaO2 < 90% and TS < 90%) changed significantly with the degree of OSA severity (*p* < 0.001). Particularly, patients with more severe OSA displayed a greater number of nocturnal desaturations, a lower average and minimum oxyhaemoglobin saturation values and they spent more time with saturation values less than 90%.

**Table 1:** Anthropometric characteristics, comorbidities, polysomnographic parameters, biochemical data and liver fibrosis score in 334 patients with obesity, divided according to OSA severity

		No OSA (*n* = 57)	Mild OSA (*n* = 88)	Moderate OSA (*n* = 65)	Severe OSA (*n* = 124)	*P*
Anthropometric data	Sex (M/F)	12/45	28/60	33/32	85/39	< 0.001
	Age (y)	41.1 ± 14.1	48.7 ± 10.6	50.2 ± 10.8	49.8 ± 12.2	< 0.001
	Weight (Kg)	114 (100.4–130.6)	115.8 (102.2–133)	122.9 (108–150.4)	132 (116.2–154.8)	< 0.001
	BMI (Kg/m^2^)	40.7 (36–47.5)	41 (37.4–47.9)	44.3 (40.1–49.2)	45.2 (39.6–51.2)	< 0.001
Comorbidities	T2DM	10 (17.5)	25 (28.4)	23 (35.4)	43 (34.7)	0.079
	Prediabetes	8 (14)	18 (20.4)	10 (15.4)	18 (15.3)	0.645
	Hypertension	21 (36.8)	50 (56.8)	39 (60)	94 (75.8)	< 0.001
	Dyslipidemia	23 (40.3)	53 (60.2)	43 (66.1)	78 (62.9)	0.015
Polysomnographic data	mSaO2%	87 (81–91)	84 (77–87)	78 (69–83)	68 (61–76)	< 0.001
	meanSaO2%	95 (94–96)	94 (93–95)	93 (92–95)	92 (89–93)	< 0.001
	nSaO2 < 90%	0.1 (0.0–0.6)	1.4 (0.5–4. 175)	6 (2.60–9.30)	25.1 (12.2–19.6)	< 0.001
	TS < 90%	0.1 (0.0–1.6)	1.2 (0.3–5.1)	5.2 (1.7–13.8)	30.6 (12.3–48.2)	< 0.001
Biochemical data	TC (mg/dL)	175 (152–190)	187 (156–209)	187 (163–221)	176 (154–206)	0.187
	LDL (mg/dL)	99 (89–128)	118 (87–138)	113 (96–138)	113 (89–138)	0.152
	HDL (mg/dL)	49 (38–56)	46 (39–57)	42 (37–51)	41 (34–48)	< 0.001
	TG (mg/dL)	106 (67–133)	103 (73–133)	120 (95–197)	120 (87–165)	0.008
	Vitamin D3 (nmol/L)	36 (22–52)	35 (23–54)	27 (19–42)	27 (18–40)	0.012
	FPG (mmol/L)	5.2 (5–5.8)	5.7 (5.-6.6)	5.8 (5.1–7.3)	5.8 (5.3–7)	< 0.001
	Insulin (mU/L)	12.7 (8.5–24)	14.3 (9.6–22.5)	20 (13–30)	22.2 (16–36)	< 0.001
	HOMA	2.8 (1.8–5.8)	4 (2.3–5.8)	5.3 (3.1–8.8)	6 (4–8.9)	< 0.001
	Platelets (× 10^9^/L)	257 (213–311)	236 (211–295)	242 (208–306)	238 (190–269)	0.042
	ALT (U/L)	23 (16–31)	23 (18–37)	26 (20–33)	29 (21–40)	0.016
	AST (U/L)	23 (18–32)	23 (19–32)	26 (21–32)	30 (24–42)	< 0.001
	GGT (U/L)	21 (14–37)	21 (14–40)	29 (18–44)	30 (22–44)	< 0.001
	Leptin (ug/L)	33 (22–48)	36 (20–50)	34 (21–47)	31 (19–44)	0.661
	hs-CRP (mg/L)	3.94 (1.95–6.88)	4.36 (2.41–7.14)	4.14 (2.91–7.65)	4.72 (3.24–6.73)	0.912
	IL-6 (ng/L)	1.9 (1.9–3.28)	2.1 (1.9–3.1)	2.4 (1.9–4.1)	2.6 (1.9–3.9)	0.024
	TNF-α (ng/L)	7.1 (5.85–8.6)	6.8 (5.6–9.5)	8 (6.42–9.4)	7.7 (6.18–9.35)	0.133
Liver fibrosis score	FIB-4	0.79 (0.48–1.26)	0.97 (073–1.42)	1.07 (0.74–1.48)	1.20 (0.91–1.85)	< 0.001

*M* male, *F* female, *BMI* Body Mass Index, *T2DM* Type 2 Diabetes Mellitus, *mSaO2%* minimum percentage oxyhaemoglobin saturation (SaO2) %, *nSaO2 < 90%* number of desaturations with a SaO2 < 90%, *TS < 90%* the percentage of time with SaO2 < 90%, *TC* total cholesterol, *LDL* Low Density Lipoprotein–cholesterol, *HDL* High-Density Lipoprotein–cholesterol, *TG* triglycerides, *FPG* Fasting plasma glucose, *HOMA* Homeostasis model assessment-insulin-resistance index, *ALT* alanine aminotransferase, *AST* aspartate aminotransferase, *GGT* gamma glutamyl transferase, *hs-CRP* high-sensitivity C-Reactive Protein, *TNF-a* Tumor Necrosis Factor-a, *IL-6* interleukin-6, *FIB-4* Fibrosis-4 index

Statistical analysis between the four groups was performed with One Way Analysis of Variance when Normality Test (Shapiro–Wilk) and Equal Variance Test (Brown–Forsythe) have been passed (data are presented as mean values ± standard deviations) or, if not, with the Kruskal–Wallis One Way Analysis of Variance on Ranks (data are presented as median value (25th–75th percentile)) and with Chi-square test in categorical variables; results were reported in the p column

The progression versus higher levels of OSA severity was associated with male (*p* < 0.001), age (*p* < 0.001), weight (*p* < 0.001), presence of hypertension (*p* < 0.001) and dyslipidemia (*p* = 0.015); even though not statistically significant, the percentage of patients with diabetes was gradually higher going through OSA classes. Consensually, the more severe OSA the higher were FPG (*p* < 0.001), insulin (*p* < 0.001), and HOMA (*p* < 0.001) levels. Moreover, HDL and TG levels significantly decreased (*p* < 0.001) and increased (*p* = 0.008), respectively, with OSA severity. We found that only IL-6 (*p* = 0.024) and not hs-CRP and TNF-a, was associated with OSA severity. Interestingly, also Vitamin D3 levels were statistically different between OSA classes.

**Fig. 1:**
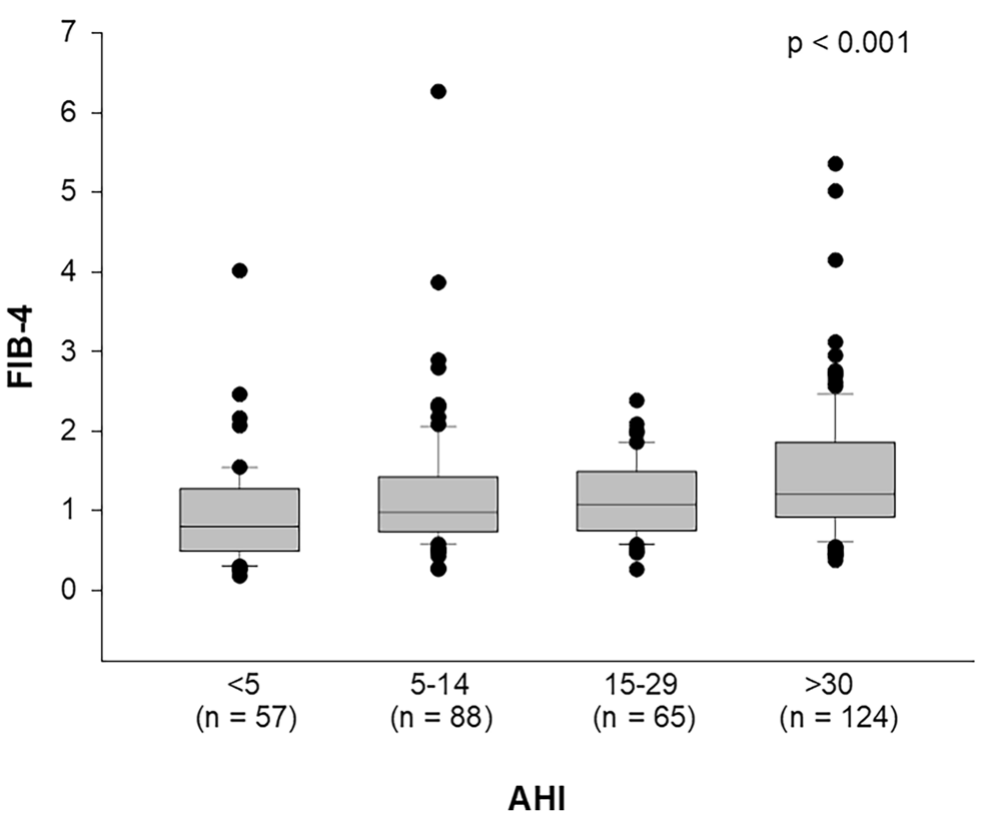
Association of FIBrosis-4 index (FIB-4) with severity of Obstructive Sleep Apnea, determined by Apnea Hypopnea Index (AHI). Results are reported as box plot graphs: the box represents the lower and upper quartiles, the line in the box represents the median, the whiskers show the lowest and highest values, and the outliers are represented by black circles. Statistical analysis was performed by the Kruskal–Wallis One Way Analysis of Variance on Ranks

Confirming the association between NAFLD and OSA severity, we showed a statistically significant increase of transaminase levels (ALT *p* = 0.016, AST *p* < 0.001, GGT *p* < 0.001) and a decrease of platelets (*p* = 0.042), with OSA severity. The relationship existing among the two diseases has been demonstrated by the association between AHI and the score of liver fibrosis, FIB-4 (*p* < 0.001) (Fig. 1).

### Linear correlation analyses and multiple regression

FIB-4 values were significantly correlated with sex (male = 0, female = 1, *r* = − 0.274, *p* < 0.001), age (*r* = 0.416, *p* < 0.001), diabetes (presence = 1, absence = 0, *r* = 0.154, *p* < 0.01), dyslipidemia (*r* = 0.179, *p* < 0.01), hypertension (*r* = 0.208, *p* < 0.001), AHI (*r* = 0.148, *p* < 0.01); meanSaO2% (*r* =− 0.131, *p* < 0.05); nSaO2 < 90% (*r* = 0.225, *p* < 0.01), BMI (− 0.169, *p* < 0.01), platelets (*r* =− 0.604, *p* < 0.001), AST (*r* = 0.645, *p* < 0.001), ALT (*r* = 0.341, *p* < 0.001), GGT (*r* = 0.405, *p* < 0.001), HDL (*r* = − 0.185, *p* < 0.001), TG (*r* = 0.279, *p* < 0.001), HOMA (*r* = 0.228, < 0.001), leptin (*r* = 0.212, *p* < 0.001) (Table 1, simple linear coefficients (*r*)).

To investigate the role of different polysomnographic parameters as independent predictors of liver fibrosis, we computed a multiple regression model with FIB-4 as the dependent variable and the variables found to be significantly related to FIB-4 in simple correlation analysis as independent variables. We excluded some multicollinear variables (in particular, AHI and nSaO2 < 90% because of presenting a VIF greater than 4), and transaminases, platelets and age because already included in the FIB-4 score. In this model, TG, sex, meanSaO2%, BMI and HOMA were the only variables independently correlated with REE in 334 patients with obesity (*r* = 0.47, AdjRsqr = 0.197) (Table 2).

**Table 2:** Simple linear correlation and regression analyses of FIB-4 in 334 patients with obesity

	FIB-4		
	Simple linear coefficients (*r*)	Regression coefficients (*β*)	Regression *p*
Sex^	− 0.274***	− 0.178	0.004
Age	0.416***		
Diabetes#	0.154**		
Dyslipidemia#	0.179**		
Hypertension#	0.208***		
AHI	0.148**		
meanSaO2%	− 0.131*	− 0.157	0.016
nSaO2 < 90%	0.225**		
BMI	− 0.169**	− 0.208	0.002
Platelets	− 0.604***		
AST	0.645***		
ALT	0.341***		
GGT	0.405***		
HDL	− 0.185***		
TG	0.279***	0.218	< 0.001
HOMA	0.228***	0.137	0.044
Leptin	0.212***		

*AHI* Apnea Hypopnea Index, *meanSaO2%* mean oxygen saturation %, *nSaO2 < 90%* number of desaturations with a SaO2 < 90%, *BMI* Body Mass Index, *AST* aspartate aminotransferase, *ALT* alanine aminotransferase, *GGT* gamma glutamyl transferase, *HDL* High-Density Lipoprotein–cholesterol, *TG* triglycerides, *HOMA* Homeostasis model assessment insulin-resistance index, *FIB-4* Fibrosis-4 index

Simple linear correlations were calculated by Pearson’s correlation. Regression coefficient e Regression *p* of the variables found to be independent related to FIB-4 were reported. ^male = 0, female = 1. #absence = 0, presence = 1

*p < 0.05; **p < 0.01; ***p < 0.001

## Discussion

We have investigated the relationship between the presence and severity of OSA and liver fibrosis in a large sample of patients with severe obesity undergoing polysomnography for diagnostic reasons. Consensually with literature, OSA was associated with a greater severity of obesity both in terms of BMI and metabolic complications. We demonstrated an independent correlation between the mean SaO2% values, an OSA severity index, and FIB-4 values, suggesting an independent role of this parameter in recognizing patients with greater risk of NAFLD progression towards stages characterized by growing fibrosis.

In our population the prevalence of hypertension, dyslipidemia and diabetes increased consensually with the severity of the OSA, with statistical significance for the two first, in agreement with previous studies [4, 5, 11, 40]. In particular, AHI was associated with a reduction of HDL plasma levels and with a TG increase, both parameters typically involved in the metabolic syndrome and the latter in NAFLD, suggesting an association between hypoxia and lipids accumulation. Accordingly, we observed an increase in FPG, insulin levels and the HOMA index with the increase in AHI. We could hypothesize a direct relationship between the severity of insulin resistance and the worsening of the OSA, independent of the degree of obesity, roughly determined by BMI. The treatment of OSA with CPAP is associated with an improvement in insulin resistance in the absence of significant changes in the BMI [41]. Moreover, the IL-6 values, a not specific marker of inflammation, were related to the severity of the OSA. These variations agree with the hypothesis that the low-grade inflammation plays an important pathogenetic role both in OSA-related cardiovascular complications and in the progression from fatty liver disease to NASH [33]. On the other hand, no correlation was demonstrated between IL-6 and FIB-4, probably due to the lower plasma levels of IL-6 respect to the levels in the adipose tissue.

Focusing on transaminases levels, we noticed that they were similar in the group without OSA and with mild OSA; on the other hand, in the groups with more severe degrees of OSA, a pathological increase in transaminases has been reported. These data seemed to confirm an association between worsening AHI and NAFLD, although recent guidelines have underlined that transaminases have a poor predictive value for the evolution from NAFLD to NASH [2, 3]. In patients with NAFLD, it is recommended to calculate non-invasive fibrosis markers and scores, to identify patients at higher risk to be referred to liver biopsy. In our study we have chosen to use FIB-4 whose sensitivity and specificity has been documented [26, 30]. FIB-4 were associated with the severity of the OSA. Considering the role of chronic intermittent hypoxia in the progression of metabolic liver disease [24, 33], it can be hypothesized that the indirect scores of liver disease worsen with greater number and severity of desaturations. It has been described that in pediatric NAFLD the presence of OSA is significantly associated with the presence of NASH and fibrosis, and that the severity of desaturations was related to the NASH scores and the extent of fibrosis, regardless of the degree of obesity, adiposity abdominal, metabolic syndrome and insulin resistance [42]. In children and adolescents with obesity, the degree of histologically shown fibrosis was more severe in subjects with NAFLD and OSA than in patients without respiratory syndrome. In addition, the severity and duration of nocturnal hypoxemia were associated with the progression of the histological features of NAFLD and the elevation of transaminases [43]. Finally, a direct correlation between the histological elements of NASH (degree of steatosis, degree of lobular inflammation, NAS scores and stage of fibrosis) and the presence and severity of OSA has been shown [44].

Consequently, non-invasive biomarkers and scores could become a screening tool to identify patients living with obesity and OSA to be referred to liver biopsy and therefore to prevent NAFLD progression. Indeed, could be useful to recognize those patients with OSA and higher risk to develop severe fibrosis. With this aim we focused on polysomnographic parameters which describe OSA severity. To investigate the role of different polysomnographic parameters as independent predictors of liver fibrosis, we computed a multiple regression model with FIB-4 as the dependent variable. In this model, TG, sex, meanSaO2%, BMI and HOMA were the only variables independently correlated with FIB-4 in 334 patients with obesity. It is well known how TG and insulin resistance contribute to pathogenesis to NAFLD [22], that NAFLD prevalence and severity are greater in male and that there is a strong association between NAFLD and BMI [1, 19]. Conversely, we described an inverse correlation between the two variables FIB-4 and BMI. This may be explained by the so-called “obesity paradox” where patients with obesity and cirrhosis have lower mortality than normal weight patients with cirrhosis [45]. Interestingly, we demonstrated that the meanSaO2% and not AHI, among polysomnographic parameters, is an independent determinant of FIB-4. A possible explanation may be that AHI quantifies the number of apnea episodes during the polysomnographic recording, without considering severity. The average saturation, although related to AHI values, considers the severity of desaturation events and is determined not only by the number, but also by the duration and depth of the desaturations. Thus, meanSaO2% may represent a more precise parameter to describe and correlate OSA chronic intermittent hypoxia to liver fibrosis scores.

Our study has some limitations. Firstly, we enrolled patients affected by severe obesity in a center where bariatric surgery is the main suggested treatment. This may explain the high prevalence of obesity-related comorbidities. Furthermore, the distribution of patients in the OSA classes is not balanced. The great sample size of subjects with severe OSA is explained by the fact that polysomnography is a survey that requires a not negligible commitment of time and health resources and is therefore prescribed only for symptomatic patients or with high scores on predictive tests for OSA. Indeed, electroencephalogram is lacking, nevertheless the polysomnographic analysis was performed according to the most recent AASM criteria [37] and, thus, an appropriate method. The lack of ethnic diversity is a limitation to generalizability of our findings. Finally, we did not perform liver biopsy, but this invasive procedure cannot be extended to all patients with obesity as a screening method [26–28]. Therefore, in accordance with recent guidelines, we used fibrosis scores to stratify NAFLD patients and only patients with high scores of fibrosis undergo histological confirmations with a liver biopsy [2, 3].

In conclusion, our study shows that the presence and severity of the OSA, defined by international guidelines based on the AHI, is associated with an increased in TG values and insulin resistance, suggesting an underling common pathogenetic mechanism between OSA and NAFLD. Furthermore, we demonstrated the association of fibrosis, and OSA severity in adult patients with severe obesity, confirming previous studies [32, 33, 44]. A novelty finding is that meanSaO2% is independently related to FIB-4. Therefore, meanSaO2% could play a role in recognizing patients with obesity and OSA with higher risk of developing advanced fibrosis and, thus, to undergo further investigation.

## What is already known on this subject?

- OSA correlates with NAFLD and OSA patients should be screened for the presence and severity of NAFLD.
- It is unclear the association between polysomnographic parameters and markers/scores of fibrosis.

## What this study adds?

- meanSaO2 is independently related to FIB-4, suggesting a clinical implication for recognizing patients with obesity and OSA and a higher risk of developing advanced fibrosis.
